# Supplementary material for: Iterative improvement in the automatic modular design of robot swarms
Source: PeerJ Comput Sci. 2020 Dec 7;6:e322. doi: 10.7717/peerj-cs.322 (PMC7924708; doi:10.7717/peerj-cs.322)
Supplement: Supplemental Information 3 [file peerj-cs-06-322-s003.zip › argos3/doc/api/standalone/a00478_source.html]

ARGoS: plugins/robots/foot-bot/simulator/footbot\_turret\_entity.cpp Source File


- Main Page
- Related Pages
- Namespaces
- Classes
- Files

- File List
- File Members

# plugins/robots/foot-bot/simulator/footbot\_turret\_entity.cpp

Go to the documentation of this file.

```
00001 
00007 #include "footbot_turret_entity.h"
00008 #include <argos3/core/utility/math/angles.h>
00009 #include <argos3/core/simulator/physics_engine/physics_engine.h>
00010 #include <argos3/core/simulator/space/space.h>
00011 
00012 namespace argos {
00013 
00014    /****************************************/
00015    /****************************************/
00016 
00017    CFootBotTurretEntity::CFootBotTurretEntity(CComposableEntity* pc_parent) :
00018       CEntity(pc_parent),
00019       m_psAnchor(NULL) {
00020       Reset();
00021       Disable();
00022    }
00023    
00024    /****************************************/
00025    /****************************************/
00026 
00027    CFootBotTurretEntity::CFootBotTurretEntity(CComposableEntity* pc_parent,
00028                                               const std::string& str_id,
00029                                               SAnchor& s_anchor) :
00030       CEntity(pc_parent, str_id),
00031       m_psAnchor(&s_anchor) {
00032       Reset();
00033       Disable();
00034    }
00035    
00036    /****************************************/
00037    /****************************************/
00038 
00039    void CFootBotTurretEntity::Init(TConfigurationNode& t_tree) {
00040    }
00041 
00042    /****************************************/
00043    /****************************************/
00044 
00045    void CFootBotTurretEntity::Reset() {
00046       m_unMode = MODE_OFF;
00047       m_cDesRot = CRadians::ZERO;
00048       m_cOldRot = CRadians::ZERO;
00049       m_fDesRotSpeed = 0.0;
00050       m_fCurRotSpeed = 0.0;
00051       if (m_psAnchor) {
00052          m_psAnchor->OffsetOrientation = CQuaternion();
00053       }
00054    }
00055 
00056    /****************************************/
00057    /****************************************/
00058 
00059    void CFootBotTurretEntity::Update() {
00060       /* Calculate rotation speed */
00061       CRadians cZAngle, cYAngle, cXAngle;
00062       m_psAnchor->OffsetOrientation.ToEulerAngles(cZAngle, cYAngle, cXAngle);
00063       m_fCurRotSpeed =
00064          NormalizedDifference(cZAngle, m_cOldRot).GetValue() *
00065          CPhysicsEngine::GetInverseSimulationClockTick();
00066       /* Save rotation for next time */
00067       m_cOldRot = cZAngle;
00068    }
00069 
00070    /****************************************/
00071    /****************************************/
00072 
00073    CRadians CFootBotTurretEntity::GetRotation() const {
00074       CRadians cZAngle, cYAngle, cXAngle;
00075       m_psAnchor->OffsetOrientation.ToEulerAngles(cZAngle, cYAngle, cXAngle);
00076       return cZAngle;
00077    }
00078 
00079    /****************************************/
00080    /****************************************/
00081 
00082    Real CFootBotTurretEntity::GetRotationSpeed() const {
00083       return m_fCurRotSpeed;
00084    }
00085 
00086    /****************************************/
00087    /****************************************/
00088 
00089    const CRadians& CFootBotTurretEntity::GetDesiredRotation() const {
00090       return m_cDesRot;
00091    }
00092 
00093    /****************************************/
00094    /****************************************/
00095 
00096    Real CFootBotTurretEntity::GetDesiredRotationSpeed() const {
00097       return m_fDesRotSpeed;
00098    }
00099 
00100    /****************************************/
00101    /****************************************/
00102 
00103    void CFootBotTurretEntity::SetDesiredRotation(const CRadians& c_rotation) {
00104       m_cDesRot = c_rotation;
00105       m_cDesRot.SignedNormalize();
00106    }
00107 
00108    /****************************************/
00109    /****************************************/
00110 
00111    void CFootBotTurretEntity::SetDesiredRotationSpeed(Real f_speed) {
00112       m_fDesRotSpeed = f_speed;
00113    }
00114 
00115    /****************************************/
00116    /****************************************/
00117 
00118    REGISTER_STANDARD_SPACE_OPERATIONS_ON_ENTITY(CFootBotTurretEntity);
00119 
00120    /****************************************/
00121    /****************************************/
00122 
00123 }
```

---

Generated on 10 Jul 2018 for ARGoS by 
 1.6.1
